# Skin Cancer Image Classification Using Artificial Intelligence Strategies: A Systematic Review

**DOI:** 10.3390/jimaging10110265

**Published:** 2024-10-22

**Authors:** Ricardo Vardasca, Joaquim Gabriel Mendes, Carolina Magalhaes

**Affiliations:** 1 ISLA Santarem, Rua Teixeira Guedes 31, 2000-029 Santarem, Portugal; 2 Instituto de Ciência e Inovação em Engenharia Mecânica e Engenharia Industrial, Universidade do Porto, 4099-002 Porto, Portugal; jgabriel@fe.up.pt (J.G.M.); up201607752@edu.fe.up.pt (C.M.); 3 Faculdade de Engenharia, Universidade do Porto, 4099-002 Porto, Portugal

**Keywords:** classification, machine learning, non-invasive, imaging methods, skin cancer

## Abstract

The increasing incidence of and resulting deaths associated with malignant skin tumors are a public health problem that can be minimized if detection strategies are improved. Currently, diagnosis is heavily based on physicians’ judgment and experience, which can occasionally lead to the worsening of the lesion or needless biopsies. Several non-invasive imaging modalities, e.g., confocal scanning laser microscopy or multiphoton laser scanning microscopy, have been explored for skin cancer assessment, which have been aligned with different artificial intelligence (AI) strategies to assist in the diagnostic task, based on several image features, thus making the process more reliable and faster. This systematic review concerns the implementation of AI methods for skin tumor classification with different imaging modalities, following the PRISMA guidelines. In total, 206 records were retrieved and qualitatively analyzed. Diagnostic potential was found for several techniques, particularly for dermoscopy images, with strategies yielding classification results close to perfection. Learning approaches based on support vector machines and artificial neural networks seem to be preferred, with a recent focus on convolutional neural networks. Still, detailed descriptions of training/testing conditions are lacking in some reports, hampering reproduction. The use of AI methods in skin cancer diagnosis is an expanding field, with future work aiming to construct optimal learning approaches and strategies. Ultimately, early detection could be optimized, improving patient outcomes, even in areas where healthcare is scarce.

## 1. Introduction

Skin cell mutations are common when the human body is repeatedly exposed to external hazardous situations. Ultraviolet radiation is the primary trigger for uncontrolled cell division, which can ultimately lead to the appearance of skin cancers, e.g., melanoma, squamous cell carcinoma (SCC), and basal cell carcinoma (BCC) [1,2]. The prevalence and incidence numbers associated with malignant skin tumors continue to increase, along with deaths, a tendency that can be reversed if changes are made to current detection strategies [3].

The diagnosis of skin tumors is based mainly on heuristic methods, as dermatologists try to improve their diagnosis skills through the assessment of multiple skin lesion patterns over time [4]. To assist in the visualization of characteristic features, physicians usually utilize dermoscopy devices to highly amplify the skin lesion and display features invisible to the unaided eye [5]. If a suspicion of malignancy is present, histopathological tests are prescribed and the lesion is excised/incised to reach a final conclusion [6]. Apart from being painful and sometimes risky for the patient, the current detection strategy is highly biased, being affected by inter- and intra-observer variability [7]. To counteract this, different non-invasive imaging techniques have appeared throughout the years, in the hopes of increasing the accuracy of early diagnosis while avoiding the performance of needless biopsies. Microscopy-based techniques, such as confocal scanning laser microscopy and multiphoton laser scanning microscopy, are some of the options preferred to dermoscopy equipment. The former scans samples with a single-photon laser, producing a three-dimensional (3D) image from a compilation of multiple two-dimensional (2D) images retrieved at different depths. The latter utilizes multiple photons to excite fluorescent molecules, delivering 3D images of deeper structures with great resolution. Still, the implementation of technologies based on infrared thermal (IRT) imaging, where temperature maps are produced based on the emission of infrared radiation, and ultrasonography, where images are produced based on the reflection of soundwaves, can also be found [8,9].

While the use of alternative techniques can provide valuable and unique knowledge, the interpretation of the collected data can not only be a tedious task but extremely hard to perform, due to the number of generated points, especially with a growing patient flow.

To address these challenges, artificial intelligence (AI) offers promising solutions.

AI is a relevant strategy for improving image analysis, reducing the time required and improving sensibility and accuracy [10]. Thus, many AI strategies based on machine learning (ML) methods have been developed. These strategies deliver a final diagnosis based on a set of input features that can potentially help domain experts to decide on the best course of action; this ultimately contributes to better patient care in a faster and cheaper way [11,12].

This work presents a systematic review focused on the critical evaluation of the implementation of AI methods for skin tumor classification, offering insights into their effectiveness across different imaging modalities.

## 2. Methods

The Preferred Reporting Items for Systematic reviews and Meta-Analyses (PRISMA) 2020 guidelines were followed to perform the present systematic review [13]. Studies focused on the application of AI classifiers for skin cancer classification through imaging data were meticulously chosen and evaluated, with the collected data partially employed to produce conclusions based on statistical methods. This review was registered on the PROSPERO registration system with the reference number 584122.

### 2.1. Information Sources

Three abstract and citation databases were used during the literature search process: PubMed, ISI Web of Science, and Scopus. The specific combination of keywords and Boolean operators implemented was as follows: (classification methods [Title/Abstract]) AND (imaging [Title/Abstract]) AND (artificial intelligence) AND (skin cancer [Title/Abstract]) OR (skin neoplasm/Abstract]); TOPIC: (classification methods) AND TOPIC: (imaging) AND TOPIC: (artificial intelligence) AND TOPIC: (skin cancer OR (skin neoplasms)); (TITLE-ABS-KEY (classification methods) AND TITLE-ABS-KEY (imaging) AND TITLE-ABS-KEY (artificial intelligence) AND TITLE-ABS-KEY (skin cancer OR (skin neoplasms)). These sources were consulted until June 2024. The main goal was to gather a large number of articles referring to the implementation of classification methods for skin cancer identification with a given imaging modality by using simple and broad terms. The Boolean operator OR was used for the similar expressions “skin cancer” and “skin neoplasm”, since different terminology is applied when referring to skin tumors. Duplicate articles were removed after bibliographic research.

### 2.2. Eligibility Criteria and Screening

After searching reference sources, the titles and abstracts of the retrieved records were screened to eliminate studies that did not focus on the topic of interest. Seven elimination criteria were established. Articles that performed classification using methods that did make use of ML were excluded, as well as studies that implemented AI methods only for image analysis tasks. The third eligibility criterion removed reviews and meeting abstracts. Papers written in languages other than English or those focused on the diagnosis of other skin diseases were also not considered. Additionally, only articles that reported the performance metrics of classification methods were retained; reports focused on the development of smartphone apps were also eliminated.

## 3. Results

### 3.1. Study Selection

**Figure 1:**
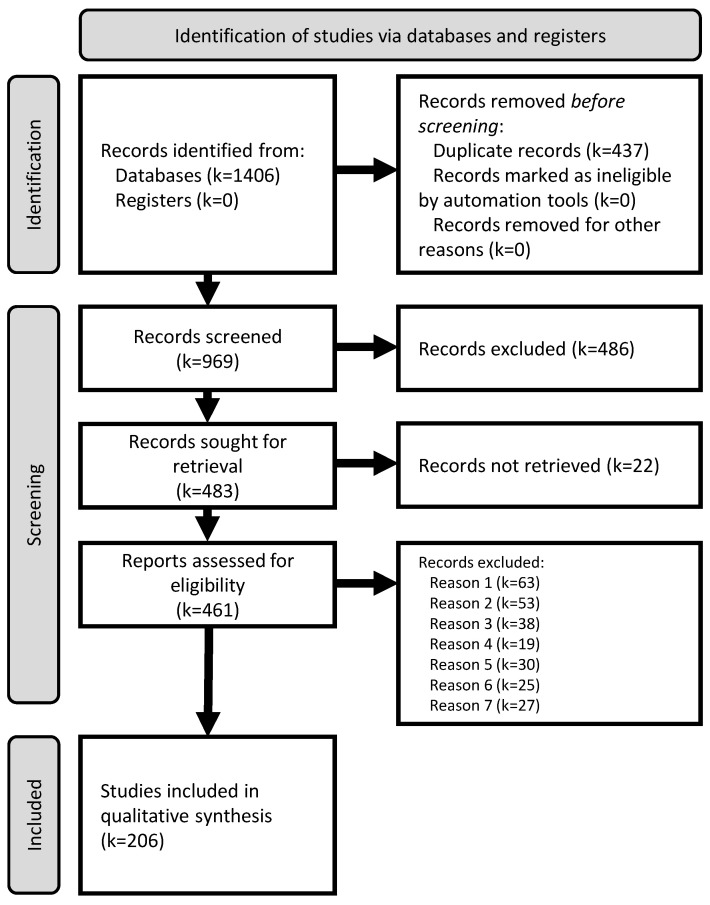
PRISMA 2020 flow diagram summarizing the review process (based on [13]).

The PRISMA flow diagram in Figure 1 illustrates the selection process [13]. The database search yielded 1406 publications. After removing duplicate records, 437 records were removed from the pool. The title and abstract screening resulted in the exclusion of 486 records because there was no mention of AI classification methods being used for skin cancer images, and 22 records could not be retrieved. An additional 255 studies were eliminated for the following reasons: making no use of ML algorithms for classification (*n* = 63), the implementation of AI strategies only for image analysis (*n* = 53), being meeting, abstracts, or literature reviews (*n* = 38), being written in a language other than English (*n* = 19), focusing on the diagnosis of other skin diseases (*n* = 30), not presenting any type of classification metrics (*n* = 25), and focusing on the development of smartphone apps (*n* = 27).

The remaining 206 records were subjected to qualitative assessment and divided into groups based on the imaging modality implemented for skin tumor assessment, as follows: digital dermoscopy (*n* = 153), microscopy (*n* = 19), spectroscopy (*n* = 12), macroscopy (*n* = 17), and other imaging modalities (*n* = 5). Table 1 summarizes the records encountered, according to image modality.

### 3.2. Qualitative Synthesis

#### 3.2.1. Digital Dermoscopy Imaging

The use of dermoscopy as the gold standard for assessing melanocytic lesions has increased the attention of researchers focused on the development of computer-aided diagnostic tools. Thus, most studies found on the subject of ML applications for skin cancer are based on this imaging modality.

The support vector machines (SVM) algorithm is consistently used for a fast and effective interpretation of data in melanoma detection studies, combined with different image analysis strategies and training stages [14,15,16,17,18,19,20,21,22]. Vasconcelos et al. proposed a methodology that extracts 660 color descriptors and detects melanoma skin cancer using SVM with a 3rd order polynomial kernel [23]. The use of textural features has also been reported with an emphasis on local binary patterns (LBP) and gray level co-occurrence matrix [24]. Khan et al. developed an algorithm to improve lesion segmentation accuracy, by enhancing the contrast between the lesion’s foreground and background. Feature selection was performed using a genetic algorithm, that best differentiated melanoma and melanocytic nevus with an SVM learner [25]. Excellent results have also been previously achieved by the same group after performing feature selection by calculating the Bhattacharyya distance and variance and subsequent lesion classification with a multi-class SVM (accuracy (ACC) = 98.3%, sensitivity (SN) = 97.85, specificity (SP) = 100%) [26]. High ACC was also achieved by Rehman et al., who constructed a method with pixel-based seed segmentation and the concatenation of multilevel features that allowed for the detection of malignant melanocytic lesions with a 98.2% success rate [27]. Massod et al. followed a different strategy and upgraded a training stage with a self-advised SVM to learn from data that are not linearly separable, which outperformed common SVM learners [28]. The same concept was applied further, using a self-advised SVM in parallel with three independent classifiers to boost the results, but with slightly lower metrics than those previously attained (ACC = 89%, SN = 90%, SP = 88.3%) [29]. Frequently, the success of the proposed methodology is hampered by the use of datasets that misrepresent one or more classes. Thus, some techniques to deal with class imbalance are often encountered, such as random under sampling, random under sampling boosting, and synthetic-minority over sampling techniques, with the latter being favored [30,31,32]. Recently, support vector learners have also been applied for the detection of other skin lesion types, such as BCC and SK, as well as for the identification of lesions as candidates for excision or not [33,34,35,36]. Two-stage classification is also reported. At a primary research stage, Joseph et al. distinguished benign from abnormal skin lesions, with the latter being classified as dysplastic nevi or melanoma, with equally good results [37]. The successful differentiation of melanocytic and non-melanocytic tumors was reported by Suganya, followed by separating non-melanocytic lesions into BCC and AK [38]. Some authors have demonstrated that the use of SVM ensemble methods provides superior results to those of a single learner for this type of classification task, with a two-stage classification approach yielding better results than a single multi-classification stage [39,40,41,42,43,44,45]. The use of different feature subsets as inputs for the learning algorithms was also proven to boost classification results [40,43], with majority voting, decision template combination, and sum rules as examples to determine the final consensus [39,40,42,44].

Artificial neural networks (ANNs) for skin cancer classification have gained particular attention recently [46,47,48,49]. Messadi et al. detected melanoma tumors with a 76.76% ACC through the implementation of a simple ANN with a hidden layer comprising four neurons [50]. The use of shape and several textural parameters allowed the attainment of good results, similar to those of healthcare physicians, with a very straightforward approach. With a more elaborate view, Grochowski et al. used an ensemble of 10 neural networks to evaluate handcrafted features retrieved from melanocytic tumors. The results were combined using a single ANN, obtaining ACC, SN, and SP values of 84.4%, 90.8%, and 78%, respectively [51]. To simultaneously classify seven skin lesion types, Samsudin et al. decomposed images using a multi-resolution empirical mode into several bidimensional intrinsic mode functions (BIMFs) [52]. The BIMFs were combined with LBP features retrieved from the leased area and fed into an ANN learner, achieving an extremely high ACC value (98.9%) for multiclassification with a single learner. The search for excellent metrics has been noted in recent years, with attention shifting towards the use of deep learning techniques [53,54], particularly those focused on convolutional neural networks [55,56,57,58,59,60,61,62,63], with applications ranging from the detection of melanocytic lesions [64,65,66] and non-pigmented tumors [67] to multiple skin neoplasm types [68]. This tendency has grown increasingly during the last year [69,70,71,72,73,74,75,76,77,78,79]. Li et al. tackled the problem of class imbalance, using generative adversarial networks (GANs) to produce high-quality images of unbalanced classes [80]. The classification stage included an ensemble of convolutional neural network (CNN) models that performed multilayer feature fusion based on a fuzzy rank. Additionally, the focal and cross-entropy loss functions were combined to decrease the distance among samples that belonged to the same class, thereby improving the recognition rate of the model (ACC = 95.82%). Because these networks are usually very elaborate, Albahar proposed the use of a regularizer technique, based on the standard deviation of weights, to control the classifier’s complexity [81]. On the other hand, Kaur et al. built a CNN from scratch, and they suggested a network with multiple connect blocks organized in a manner that allowed large feature information to pass easily through the network [82]. With the need for extensive training data to properly train a network of this type, most authors chose pretrained models and performed small changes when needed [83,84,85,86,87,88]. This approach saves time, reduces computation power, and usually delivers more accurate and effective models. Hosni et al. served as an example, as the theory of transfer learning was successfully applied to a CNN with the small replacement of the classification layer with a softmax layer, which allowed for multi-classification with increased ACC values [89]. Lu et al. proposed XceptionNet with a swish activation function and depthwise separable convolutions [90]. The altered model reached the maximum ACC for the differentiation of multiple skin neoplasm types available in a public database. Recent studies have mostly focused on the use of public databases to develop different classification strategies [91,92,93,94,95,96,97,98,99]. The availability of these databases also eliminates the problem related to the shortage of large amounts of training data for the implementation of DL networks and eases comparisons between works and state-of-the-art methods. Another reported option for the improvement of CNN models is the combination of patient and image data, as demonstrated by Pacheco and Krohling, which secured an improvement of 7% in ACC compared with the single use of lesion features as inputs [100]. Based on these results, the authors used metadata to support lesion classification, emphasizing the most important feature maps extracted from images in different CNN models [101]. This strategy outperformed other approaches that did not implement metadata. Ningrum et al. compared the classification ACC of a CNN with image data input and a CNN combined with an ANN to handle image data and patients’ metadata, respectively [102]. The latter delivered better performance metrics (ACC = 92.34%) while still running on a medium-class computer. The results of other reports support the integration of clinical data as a means of reducing diagnostic uncertainty [103,104,105,106]. Despite their complexity, the intricacy of using these models for classification is valuable when dealing with equally complex tasks, such as the differentiation of multiple skin tumor types [107,108]. An attention cost-sensitive DL-based meta-classifier was proposed by Ravi with the goal of effectively detecting minimal portions of skin affected by a skin tumor and retrieving information from it to successfully detect and classify seven types of skin neoplasms (ACC = 99.0%) [109]. A different multiclass classification approach was proposed by Zhang et al. based on the extraction and input of rotation invariant features that better represent the lesion in question [110]. The proposed approach was embedded in a CNN, and although the classification results were higher than those of other rotation invariance methods, further work is still needed. In addition to classification, CNNs have also been implemented to assist with additional tasks such as segmentation, feature extraction, and feature selection [111,112,113,114,115,116,117,118,119,120,121,122,123,124,125,126,127]. However, new optimization ideas and applications are constantly being studied [128,129,130,131,132,133,134,135,136,137,138,139,140,141,142,143,144,145,146,147,148].

The detection of melanocytic lesions using dermoscopy images has also been successfully documented for classifiers based on decision trees [149]. Oliveira et al. extracted texture and color features from different color spaces, obtaining ACC, SN, and SP values of 91.6%, 87%, and 96.2%, respectively, using an optimum-path forest classifier. The good predictive performance of random forest (RF) classifiers has enabled them to outperform ensemble approaches, as proven by Rastgoo et al. [150] (SN = 94%, SP = 92%) with RF vs. weighted ensemble classifiers and Grzesiak-Kopéc et al. [151] (SN = 85.1%, SP = 89.6%) with bagging with decision tree vs. vote ensemble classifiers. The detection of BCC lesions was accomplished with an ACC of 82.4% by an RF learner after the implementation of a novel segmentation method, based on an independent component analysis, by Kharazmi et al. [152]. The same group of authors aimed to improve the classification metrics by automatically detecting the of vascular features that are characteristic of this type of lesion (SN = 90.4%, SP = 89.3%) [153].

**Figure 2:**
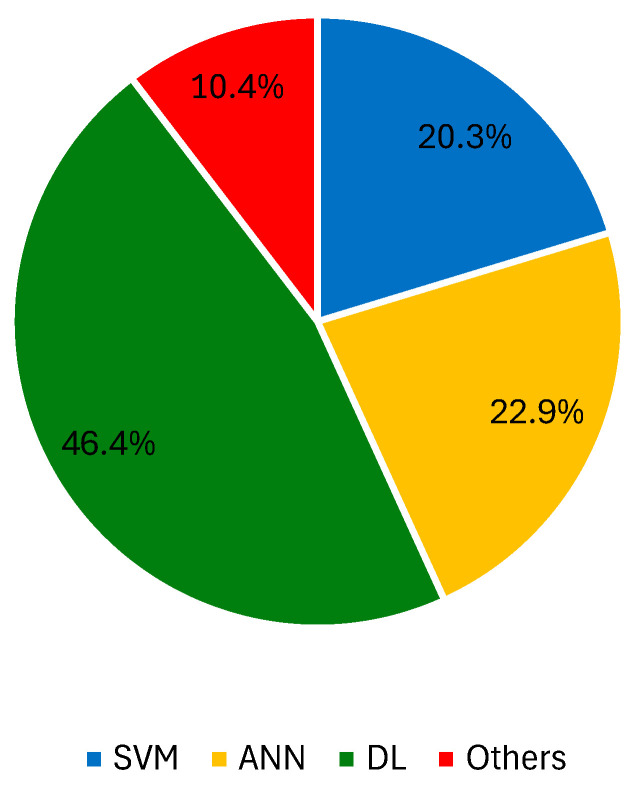
Percentage of records that used SVM, ANN, DL approaches, and other ML techniques with dermoscopy images.

When there is doubt regarding the best learner for this task, the comparison of different ML algorithms is an option [154,155]. La Torre et al. evaluated the recognition skills of k-nearest neighbor (kNN), SVM, and ANN learners. The top choice was SVM with a radial basis function kernel, which delivered SN and SP values of 100% and 99.47%, respectively. Another example of SVM’s advantage was reported in [156], where asymmetry features were collected and used for melanoma detection in dermoscopy images. This SVM technique had a 3^rd^-grade polynomial kernel better than kNN, naïve Bayes, boosting, and random forest in this task. The same learner exceeded both kNN and random forest in a new study [157]. In the differentiation of benign and melanoma lesions, conducted by Narasimhan et al. [158], RF outperformed kNN, naïve Bayes, and SVM with the use of wavelet-based energy features and outperformed kNN, SVM, ANN, and decision trees in a study by Janney and Roslin [159]. For the challenging task of distinguishing between melanoma and dysplastic lesions, RF was also considered the top learner, achieving SN and SP values of 98.46% and 70%, respectively [160]. Other methods, such as kNN [161,162] and AdaBoost [163], are also preferred. To deal with a complex and extensive dataset, HAM10000, Arshad et al. tested lesion classification with 10 different ML methods after augmentation, feature extraction, and fusion using two fine-tuned DL models (ResNet-50 and ResNet-101) [164]. A CNN with a Harris hawks optimized segmentation yielded the best outcomes (ACC = 97.3%, SN = 96.1%, SP = 98.6%) at the expense of other CNNs, ANNs, and SVM methods. Figure 2 shows the percentage of records that used SVM, ANN, DL approaches, and other ML techniques with dermoscopy images.

#### 3.2.2. Microscopy Imaging Techniques

**Figure 3:**
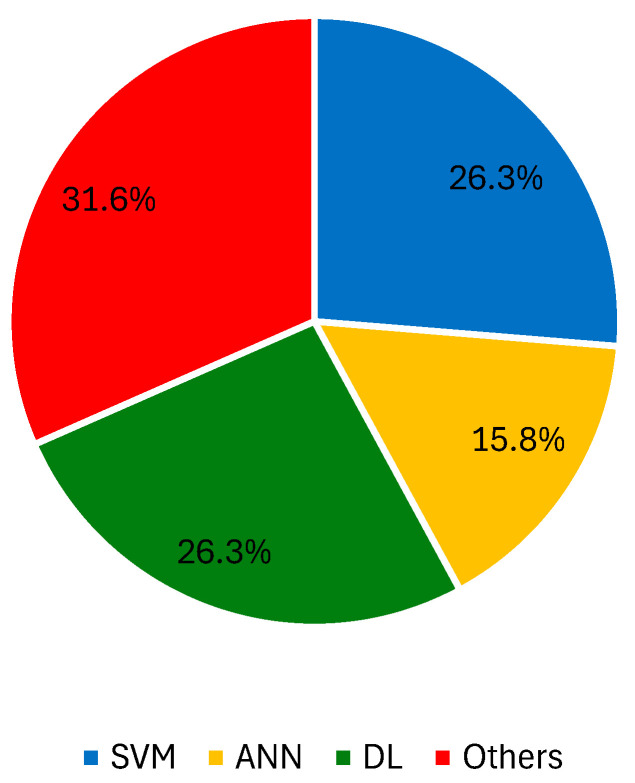
Percentage of records that used SVM, ANN, DL approaches, and other ML techniques with microscopy images.

AI classifiers have shown potential in the diagnosis of melanoma through the extraction of features from various microscopy imaging techniques [165,166,167]. The first report is from a kNN learner with 24 neighbors for a two-stage classification process involving the distinction of benign, malignant, and dysplastic nevi (SN = 87%, SP = 92%) [168]. Odeh et al. also used this ML algorithm with varying k odd numbers from one to nine to select the best possible result [169]. After achieving maximum metrics with k = 1, the authors compared the kNN learner to a k-nearest neighbor with a genetic algorithm (GA), an ANN with GA, and an adaptive neuro-fuzzy inference system, with the kNN–GA (k = 1) having the best performance [170]. The implementation of decision trees using images acquired by confocal laser scanning microscopy was also well documented by the same research group. Wavelet transform features were extracted and used for classification, obtaining SN and SP values of 97.59% and 96.32% [171,172]. Later, the same group tested a similar approach with a different training and testing set, but with unsatisfactory results (SN = 44.32% and SP = 53.29%) [173]. X-polarized and transillumination epiluminescence microscopy images enabled the extraction of textural information for the identification of malignant lesions with 70% ACC, using an SVM with a 4th order polynomial kernel [174]. The implementation of collaborative methods also yielded interesting results. Masood et al. [175] constructed a deep belief NN in parallel with an advised SVM, combining its results with least squares estimation weighting to achieve ACC, SN, and SP values of 91.6%, 95%, and 93%, respectively. Ruiz et al. [176] also combined the outcomes of different classifiers (kNN, ANN, and naïve Bayes) using a vote-based system. The best result was obtained with a k = 7 and a hidden layer with seven neurons for the kNN and ANN learners, respectively. Noroozi et al. focused on the distinction of SCC from other lesions, first using features extracted from intensity profiles (naïve Bayes, ACC = 83.3%, SN = 84.6%, SP = 81.8%) [177] and then using Z-transform coefficients (SVM, ACC = 85.18%, SN = 91.66%, SP = 80%) [178]. The use of clinical data, e.g., patient age or lesion location, to assist skin cancer diagnosis using microscopy was reported by Hohn et al. [179]. The authors tested the implementation of a CNN to investigate which strategy delivered the best results: the use of individual patient or image data, or the fusion of both. In most cases, CNN performance with only the microscopy image information was sufficient to obtain a top performance.. A CNN with gradient-weighted class activation mapping (Grad–CAM) outperformed 20 pathologists (ACC = 93.3% vs. ACC = 73.2%). The inclusion of Grad–CAM improved physicians’ trust in classification results because the saliency map showed the exact areas of images where features were extracted, which matched normally accepted histological features. Finally, some recent reports have described the implementation of Raman confocal microscopy or hyperspectral microscopy imaging, retrieving data from different image sections that are then feed to a CNN-based classifier, e.g., ResNet50 [180], U-Net [181], or a RF learner [182]. Figure 3 presents the percentage of records that used SVM, ANN, DL approaches, and other ML techniques with microscopy images.

#### 3.2.3. Spectroscopy Imaging Techniques

**Figure 4:**
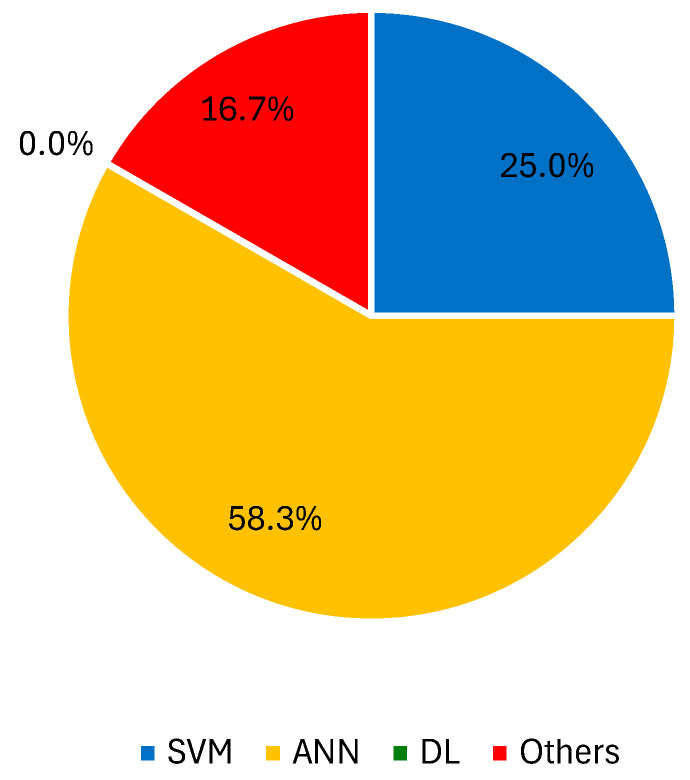
Percentage of records that used SVM, ANN, DL approaches, and other ML techniques with spectroscopy images.

The extraction of features from spectroscopy data for effective skin lesion characterization and classification has also been documented [183,184,185,186,187,188,189]. Several learners, namely ANN (half number inputs + half number outputs = six hidden units), kNN (k = 3), and naïve Bayes, were compared by Li et al. in the diagnosis of melanoma using a spectroscopy device. Statistical features were computed based on image pixel intensities, and naïve Bayes proved to be the most suited for the classification task [190]. Likewise, Maciel et al. chose to test a kNN with three neighbors to achieve the best possible distinction between BCC, psoriasis, Bowen’s disease, and skin exposed to the sun. The authors attested that the SN and SP values did not vary greatly with an increase in the number of neighbors, indicating the classifier’s stability [191]. Tomatis et al. presented a multispectral approach that collects features at different wavelengths. The first study delivered results of SN = 78% and SP = 76% with a backpropagation neural network, while the second one delivered SN and SP values of 80.4% and 90%, respectively, with a multilayer perceptron, mostly due to major improvement in the image analysis stage [192,193]. The use of an ensemble classifier for spectroscopy was mentioned by only one author [194]. Electrical impedance spectra and clinical information were used as inputs for the ANN, kNN, SVM, and partial least squares classifiers, leading to high SN but low SP values. Figure 4 presents the percentage of records that used SVM, ANN, DL approaches, and other ML techniques with spectroscopy images.

#### 3.2.4. Macroscopy Imaging

**Figure 5:**
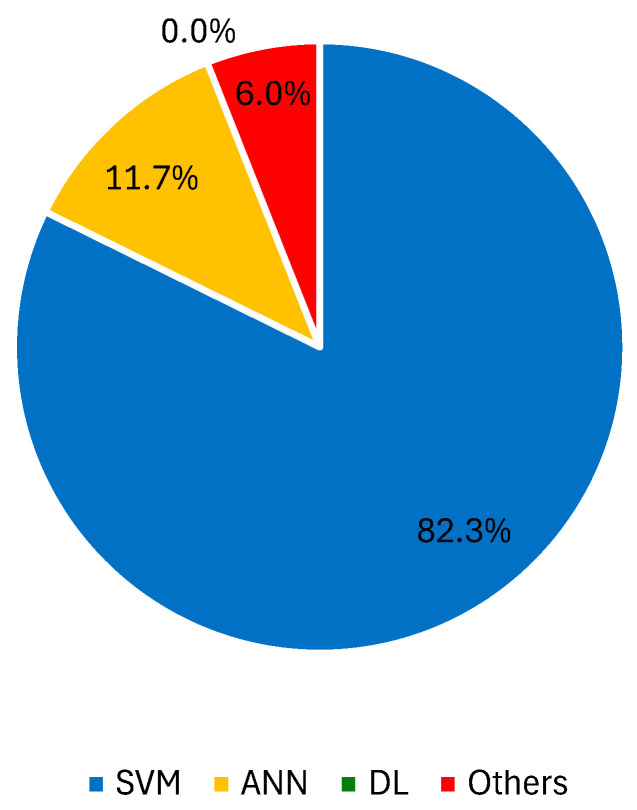
Percentage of records that used SVM, ANN, DL approaches, and other ML techniques with macroscopy images.

While there is a great tendency toward the development of AI systems for the classification of images acquired using handheld devices, some authors have focused their research on the development of CAD tools for the characterization of melanoma lesions using macro images. As in dermoscopy, SVM-based algorithms are preferred, with SN metrics ranging from 80% [195] to 100% [196], and most previous research has focused on feature extraction strategies [197]. Tabatabaie et al. [198] used an independent component analysis to extract features that described the structure and color of tumors and used a radial basis function kernel (γ = 8) for classification. The same kernel was selected by Gautam (C = 2.3784, γ = 3.3636) in an approach that counteracted the nonuniform illumination caused during image collection (ACC = 76.58%, SN = 82%, SP = 70.33%) [199]. The linear kernel preferred by Przystalski et al. [200] represented each lesion in four different color spaces to achieve an ACC of 97.44%. Similarly, Amelar et al. [201] obtained ACC, SN, and SP values of 83.59%, 96.64%, and 73.45% with the same kernel function, using high-level intuitive features to describe skin lesions. Other lesion extraction strategies for SVM classification include the top ABCD features, LBP, and 2D histograms [202,203,204,205,206]. The implementation of neural networks in macroscopy images was performed by Abbes and Sellami [207] using a random training/testing set of 66%/34%, obtaining ACC, SN, and SP values of 94%, 92.85%, and 95.45%. Jafari et al. [208] constructed a set of color features based on pigmentation intensity and fuzzy c-means clustering based on color variations. The results were good but required improvements. A set of decision trees and k-nearest neighbor learners were combined to allow melanoma detection with classification metrics close to 95% with standard camera images. Only one neighbor was used with Euclidean distance, and each tree was specialized in a feature subset [209]. Ensemble strategies have also been used in digital photography. Choudhury et al. [210] exemplified a two-stage classification, with the primary detection of melanoma lesions conducted using an SVM and extreme learning machine (ACC = 97.1%), and a differentiation between SCC, BCC, AK, and melanoma based on the best classifier of the first stage (94.18%). An ensemble comprising three SVM algorithms (radial basis function, C = 1024, and γ = 8.63 × 10^−5^) was proposed by Takruri et al. [211]. The results were combined with majority voting and probability averaging fusion to obtain an ACC of 82.5%. Figure 5 shows the percentage of records that used SVM, ANN, DL approaches, and other ML techniques with macroscopy images.

#### 3.2.5. Other Imaging Modalities

**Figure 6:**
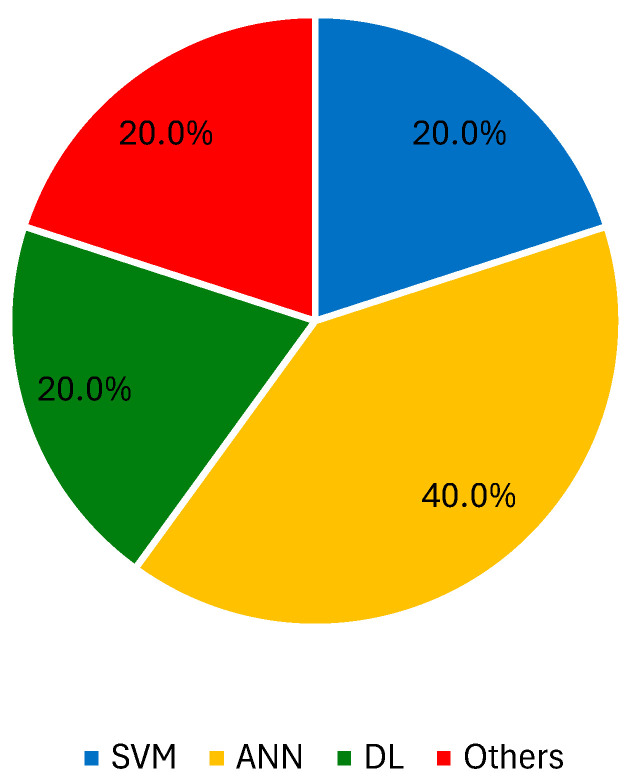
Percentage of records that used SVM, ANN, DL approaches, and other ML techniques with other imaging modalities.

The classification of skin tumors has also been performed using other imaging modalities in conjunction with ML. The use of infrared thermal imaging was explored by Magalhaes et al. [212], where thermal features retrieved from static and dynamic thermal images were computed and used as inputs to test the classification ability of different learners. The best results for the distinction of melanoma and nevi were obtained using SVM. Due to the shortage of thermal image data, the same research group experimented with different data augmentation strategies to successfully implement a DL approach and increase the previously achieved diagnostic accuracy [213]. The intensity, standard deviation, and entropy of regions of interest in sonogram images were retrieved and used for melanoma, BCC, and benign lesion distinction by Kia et al. [214]. The authors implemented a multilayer perceptron (Log-sigmoid transfer function (layer 1) and hyperbolic sigmoid transfer function (layer 2 and 3)) to achieve the maximum SN value (98%), but with a very low SP value (5%). Ding et al. [215] combined 3D and 2D ABCD features of images collected using a photometric stereo device. The SVM with a multilayer classification kernel obtained ACC, SN, and SP results of 87.8%, 94%, and 83.3%, respectively. More recently, Faita et al. [216] presented the differentiation of melanoma and melanocytic nevus using ultrasonographic images. Morphological parameters and texture and color features delivered the best classification results with a k-nearest neighbor learner (ACC = 76.9%, SN = 84%, and SP = 70%). Thus, this represents another non-invasive approach. Figure 6 presents the percentage of records that used SVM, ANN, DL approaches, and other ML techniques with macroscopy images.

## 4. Discussion

It is clear from the results encountered in the qualitative analysis that, regardless of the selected imaging modality, most classification tasks have been performed by SVM and/or ANN algorithms, with a recent focus on CNNs, e.g., [100,101,102,107,108,109,110,111,112,120]. Other learners, such as kNN, decision trees, and AdaBoost, have also been explored, but with less emphasis. When there is doubt regarding the best ML method for the proposed classification task, a comparison of different types of learners is a valid strategy frequently used to yield the best results, e.g., [23,39,151,156,158,160,170,176,190,200]. To improve the accuracy of this prediction, some studies have implemented more elaborate strategies based on ensemble models, to the detriment of single learners, e.g., [39,40,41,42,43,44,45,175,176,194,195]. The use of deep learning-based methods is also common; however, such methods involve the availability of a large dataset of labeled data [28,29,36,53,55,64,67,81,100,111,112,120,121,122,125,128,175,204]. In fact, most authors have emphasized the importance of freely available databases (e.g.: ISIC 2016, 2017, 2018, PH2, DermIS, MED-NODE) and the full disclosure of the dataset used, in order to allow the comparison of the developed classifier and/or image analysis process with those from the literature [25,26,27,30,31,32,34,36,37,51,53,54,55,81,89,111,112,120,121,122,125,150,159,163,192,193].

A detailed description of the classification parameters and training and testing conditions is scarce in some studies, likely due to the use of standard models available in ML libraries. Based on the results, the most popular tool for image analysis and classification was MATLAB, a non-open-source software [22,25,26,28,38,54,111,125,128,165,183,191,196,200,208,215], with only a few authors developing their work on free open-source platforms. Thus, to facilitate the implementation of CAD systems in daily clinical practice, the use of open-source tools is favored.

Most studies have focused on the optimization and innovation of the feature extraction stage, suggesting that it is probably more determinant than the development and/or selection of the optimal classification approach [23,39,45,168,174,195,201,204,209,211]. Different results can be achieved with the use of the same test samples; thus, its development appears to be a future trend.

Some studies do not include more than one parameter representative of learners’ performance, which hampers the comparison between different studies [27,39,40,46,100,112,174,184,200,210]. However, once again, it is important to take these results lightly, because the datasets used can differ between studies.

Lastly, it is important to guarantee an even balance regarding the correct identification of benign cases (SP) and cancerous tumor (SN) [50,55,121,159,194,212,214]. Thus, the loopholes of a given imaging modality can be compensated for by merging the different types of information. The establishment of standard metrics to evaluate the performance of classifiers is of interest, as it allows some degree of comparison. Table 2 shows the summary of the main findings encountered and their respective references.

## 5. Conclusions

The use of AI strategies based on ML methods is increasingly common in skin cancer assessment using characteristic image features. This review presents multiple noninvasive imaging modalities combined with AI. There is a clear preference for the use of SVM and ANNs, whether in a solo implementation or in an ensemble method, because of their good results with lower computational costs; deep learning approaches have also gained ground because of the exceptional classification metrics associated with their use. The optimization and innovation of the feature extraction stage is a clear topic for future work in most records because it appears to be the key to improving diagnostic accuracy. Additional testing and the comparison of different methodologies using the same dataset are of importance because they would benefit research in this area. Thus, the construction of databases freely available for scientific purposes with skin cancer images of different imaging modalities is of extreme interest for future work. Ultimately, the use of AI methods for skin cancer diagnosis is an expanding field, with accurate early detection being the primary approach used to improve patient prognosis [39,45,168].

## Figures and Tables

**Table 1:** Reference of records included in the systematic review, grouped by imaging modality.

Imaging Modality	Reference of Records
Dermoscopy	[14,15,16,17,18,19,20,21,22,23,24,25,26,27,28,29,30,31,32,33,34,35,36,37,38,39,40,41,42,43,44,45,46,47,48,49,50,51,52,53,54,55,56,57,58,59,60,61,62,63,64,65,66,67,68,69,70,71,72,73,74,75,76,77,78,79,80,81,82,83,84,85,86,87,88,89,90,91,92,93,94,95,96,97,98,99,100,101,102,103,104,105,106,107,108,109,110,111,112,113,114,115,116,117,118,119,120,121,122,123,124,125,126,127,128,129,130,131,132,133,134,135,136,137,138,139,140,141,142,143,144,145,146,147,148,149,150,151,152,153,154,155,156,157,158,159,160,161,162,163,164]
Microscopy	[165,166,167,168,169,170,171,172,173,174,175,176,177,178,179,180,181,182]
Spectroscopy	[183,184,185,186,187,188,189,190,191,192,193,194]
Macroscopy	[195,196,197,198,199,200,201,202,203,204,205,206,207,208,209,210,211]
Other imaging modalities	[212,213,214,215,216]

**Table 2:** Summary of the discussed main findings and their respective references.

Main Findings	Example of Records
CNNs are a current tendency	[100,101,102,107,108,109,110,111,112,120]
Ensembles are sometimes preferred for better ACC	[39,40,41,42,43,44,45,175,176,194,195]
Different learners can be tested to achieve best performance	[23,39,151,156,158,160,161,170,176,190,200]
Freely available databases are of extreme importance for comparison of works	[25,26,27,30,31,32,34,36,37,51,53,54,55,81,89,111,112,120,121,122,125,150,159,162,192,193]
Some authors still prefer the use of licensed software	[22,25,26,28,38,54,111,125,128,165,183,191,196,200,208,215]
Optimization of feature extraction stage is key	[23,39,45,168,174,195,201,204,209,211]
Some studies lack reports of performance metrics	[27,39,40,46,100,112,174,184,200,210]
Good balance between SN and SP is necessary	[50,55,121,159,194,212,214]

## Abbreviations

Squamous cell carcinoma	SCC
Basal cell carcinoma	BCC
Artificial intelligence	AI
Infrared thermal	IRT
Machine learning	ML
Preferred reporting items for systematic reviews and meta-analyses	PRISMA
International database of prospectively registered systematic reviews in health and social care, welfare, public health, education, crime, justice, and international development, where there is a health-related outcome	PROSPERO
Academic citation indexing and search service, which is combined with web linking and provided by Thomson Reuters	ISI
Support vector machine	SVM
Local binary patterns	LBP
Accuracy	ACC
Sensitivity	SN
Specificity	SP
Artificial neural network	ANN
Bidimensional intrinsic mode function	BIMF
Generative adversarial network	GAN
Convolutional neural network	CNN
Deep learning	DL
Random forest	RF
k-nearest neighbor	kNN
Computer aided diagnostic	CAD
Genetic algorithm	GA
Gradient-weighted class activation mapping	Grad-CAM
